# Determination of Antioxidants by DPPH Radical Scavenging Activity and Quantitative Phytochemical Analysis of *Ficus religiosa*

**DOI:** 10.3390/molecules27041326

**Published:** 2022-02-16

**Authors:** Siddartha Baliyan, Riya Mukherjee, Anjali Priyadarshini, Arpana Vibhuti, Archana Gupta, Ramendra Pati Pandey, Chung-Ming Chang

**Affiliations:** 1 Centre for Drug Design Discovery and Development (C4D), SRM University, Delhi-NCR, Rajiv Gandhi Education City, Sonepat 131029, Haryana, India; s4.sidsworld@gmail.com (S.B.); riya.mukherjee1896@gmail.com (R.M.); anjali.p@srmuniversity.ac.in (A.P.); arpana.v@srmuniversity.ac.in (A.V.); archana.g@srmuniversity.ac.in (A.G.); 2 Master & Ph.D. Program in Biotechnology Industry, Chang Gung University, No. 259, Wenhua 1st Rd., Guishan Dist., Taoyuan City 33302, Taiwan

**Keywords:** antioxidants, *Ficus religiosa*, 2-diphenyl-1-picrylhydrazyl (DPPH), nanotechnology, free-radical scavenging activity, nanoparticles, nano-MgO

## Abstract

The use of *F. religiosa* might be beneficial in inflammatory illnesses and can be used for a variety of health conditions. In this article, we studied the identification of antioxidants using (DPPH) 2,2-Diphenyl-1-picrylhydrazylradical scavenging activity in *Ficus religiosa*, as *F. religiosa* is an important herbal plant, and every part of it has various medicinal properties such as antibacterial properties that can be used by the researchers in the development and design of various new drugs. The 2,2-Diphenyl-1-picrylhydrazyl (DPPH) is a popular, quick, easy, and affordable approach for the measurement of antioxidant properties that includes the use of the free radicals used for assessing the potential of substances to serve as hydrogen providers or free-radical scavengers (FRS). The technique of DPPH testing is associated with the elimination of DPPH, which would be a stabilized free radical. The free-radical DPPH interacts with an odd electron to yield a strong absorbance at 517 nm, i.e., a purple hue. An FRS antioxidant, for example, reacts to DPPH to form DPPHH, which has a lower absorbance than DPPH because of the lower amount of hydrogen. It is radical in comparison to the DPPH-H form, because it causes decolorization, or a yellow hue, as the number of electrons absorbed increases. Decolorization affects the lowering capacity significantly. As soon as the DPPH solutions are combined with the hydrogen atom source, the lower state of diphenylpicrylhydrazine is formed, shedding its violet color. To explain the processes behind the DPPH tests, as well as their applicability to *Ficus religiosa* (*F. religiosa*) in the manufacture of metal oxide nanoparticles, in particular MgO, and their influence on antioxidants, a specimen from the test was chosen for further study. According to our findings, *F. religiosa* has antioxidant qualities and may be useful in the treatment of disorders caused by free radicals.

## 1. Introduction

Nanotechnology has gained popularity over recent years, and nanoparticles (NPs) are frequently regarded as the field’s basis as they are a major source of various nano-structured processes or substances. Nanomaterials are present in the environment and can be discovered by chance, or they can be created. In agriculture, structural engineering, electronics, and medicine make extensive use of metal NPs, which can be widely found in a range of other sectors of science and technology. Most metal NPs are manufactured using chemical as well as physical procedures, including reduction with hydrothermal pyrolysis, and stabilization with numerous chemical compounds that, subsequently, are responsible for a wide range of biohazards as well as environmental problems due to the toxicity of the substances involved [1]. Many types of malignancies have consistently increased ROS-sensitive signaling pathways, which are involved in cell growth/proliferation, differentiation, protein synthesis, glucose metabolism, cell survival, and inflammation.

In cellular signaling, reactive oxygen species, notably hydrogen peroxide, can operate as second messengers. Many cancer cells have a continuous increase in intrinsic creation of reactive oxygen species during malignant transformation, which preserves the oncogenic phenotype and promotes tumor development. Cancer cells use redox adaptation to enhance survival and build resistance to anticancer medicines by up-regulating anti-apoptotic and antioxidant chemicals. Little is understood about how an increase in intracellular oxidative stress levels is detected and converted into ROS-induced specific intracellular signaling that regulates antioxidant and survival gene expression.

Biological synthesis, which uses plants as well as microorganisms as a supplier of precursor substances, is developing as another sustainable and safer technique for making metal or metal oxide NPs. Extraction from plants, on the other hand, has a benefit over microorganisms in this application since it is simpler to handle and poses less of a biohazard. In addition, it decreases the price of maintaining microbial isolation as well as a culture medium, boosting cost competitiveness over the usage of microorganisms in the production process. A suitable protocol must lead to the improved control of metal/metal oxide NP forms, dimensions, and dispersivity, along with minimizing the requirement for purifying crafted NPs. It also reduces the need for high concentrations of organic solvents as well as harmful environmental nanoparticle modification. Scientists are increasingly interested in using extractions from plants in aqueous solutions to synthesize NPs, since it is a green technique and therefore has a negligible environmental impact. Such nanoparticles have been shown to be useful in a variety of sectors, including photocatalysis and biomedicine. It also has applications in catalysts, sensor systems, solid oxide fueling cells, sun protection products, biomedical imaging, biotransformation, antioxidants, and antibacterial activities because of its compact design with FRS properties [1].

Synthesis of various Fe, Ni, Cu, Ti, and Mg NPs has sparked a lot of interest in recent years because the NPs have unique properties and may be employed in a range of applications, namely detectors [2], catalysts [3], and applications of biology [4,5]. Magnesium oxide (MgO) NPs exhibit the best antibacterial property of all metal oxide NPs studied. The antibacterial action of MgO NPs, according to Leung et al. [6], may be related to the lack of reactive oxygen species (ROS). The production of reactive oxygen species (ROS) has been linked to cancer progression. Cancer cells have been shown to contain reactive oxygen species (ROS). Cancer cells are frequently exposed to high amounts of reactive oxygen species (ROS), which promote a malignant phenotype by stimulating prolonged proliferation, death evasion, angiogenesis, invasiveness, and metastasis [7,8]. Breast cancer cells produce more reactive oxygen species (ROS) than normal cells; however, cancer cells are more vulnerable to exogenous ROS, which could be a useful therapeutic target for selectively killing these cells. In fact, combining redox-active compounds with traditional therapy may be beneficial in amplifying the latter’s benefits and overcoming treatment resistance. Although intriguing, further research is needed to guarantee that this technique is safe, as increased ROS availability can cause a variety of adverse effects in normal cells. The breakdown of cell membranes is a proposed method for antibacterial action. Numerous approaches, notably sol-gel [9], sonochemistry [10], co-precipitation [11,12], and chemical oxidation [13], have been used to make MgO NPs. Water solutions or extraction from plants are the examples of green synthesis approaches that involve chemicals that are totally void of compounds that are environmentally damaging [14,15].

Free radicals are compounds or tiny pieces of particles with unpaired electrons in their molecular or atomic orbitals, or merely ROS, that also comprise numerous different oxygen species, such as hydrogen peroxide, which is a strong oxidizing functional group produced by cells throughout breathing and cell-mediated immune systems [16,17]. The bodhi tree has long held mythical, religious, and medical significance in Indian culture. Traditional Indian medicine has used its herbs for a variety of diseases. Its bark has traditionally been used to treat gonorrhea, ulcers, and skin problems as an antibacterial, antiprotozoal, antiviral, astringent, and antidiarrheal agent. Its leaves have antivenom properties, function as an antioxidant and regulate the menstrual cycle. By combining its properties with current medicine technologies, this old traditional tree can be used to cure a variety of ailments.

*F. religiosa* was chosen for the investigation because of its wide range of pharmacological activity. It is normally synthesized in the body and plays a key role in numerous cellular activities. Its excessive creation, on the other hand, causes cellular and molecular destruction, resulting in the production of a range of human health disorders, particularly malignancy [18,19]. Respiration, as well as other activities, generates a lot of free radicals, which may harm the body in many ways, resulting in the loss of function and sometimes even death. Damage caused by ROS may be reduced by using antioxidants, which are chemicals that can stop other molecules from oxidizing. Due to their potential to contribute electrons that may neutralize radical production, antioxidants are useful in lowering and preventing further damage through free-radical responses [20,21,22,23]. Several plants generally manufacture secondary metabolites such as polyphenols and flavonoids that function as antioxidants and play essential roles in a variety of biological processes [24,25,26,27,28,29,30]. A primary source of antioxidants that scavenge free radicals and fight against unwanted health problems caused by oxidative stress may thus be plants as well as natural ingredients.

Antioxidants are neutralizing chemicals that minimize oxidative damaging to biological processes by giving free radicals electrons and passing them off as harmless [31]. Free radicals are mostly associated with oxidative stress. The combination of oxygen with specific chemicals results in the generation of free radicals, and then, once created, the potential threat is the damage they may cause when they combine with essential cellular elements such as DNA and proteins, as well as the cell membrane [32]. Antioxidants react with free radicals and may stop damage before it starts by neutralizing them [33]. Secondary metabolites produced by plants include a wide range of antioxidants. As a result, the current work was conducted to investigate the free-radical scavenging capabilities of *F. religiosa* using its manufactured nano-MgO as well as the DPPH test technique [7].

The use of various plant leaf extracts, especially *Ficus religiosa*, in the process of the synthesis of metal oxide nanoparticles, especially MgO, has been widely carried out by previous researchers [7,14]. MgO NPs are generated using green synthesis, according to Siddartha et al. (2020) [14]. Plants are the most frequent biological substrate for MgO nanoparticles because they are cost-effective, environmentally acceptable, easy to process and handle, and safer than microorganisms. Furthermore, when compared to microbial-assisted MgO nanoparticle synthesis, there are fewer health risks because the solvents used to prepare plant extracts are largely distilled water and ethanol.

Plant extracts from various sections of the plant, such as the root, bark, leaves, flowers, fruit pulp, peels, and other portions, are used to make MgO nanoparticles. In theory, MgO nanoparticles are generated when a biological substrate reacts with Mg2+ salt, resulting in the creation of reduced Mg metal or a complex with the metal. The conversion of MgO nanoparticles from the respective precursor is attributed to physiologically active chemicals found in the leaf extract. It is also clear that the phytochemicals form a covalent link with the metal, which is then destroyed by heat treatment, resulting in MgO nanoparticles. NPs were created from the leaves of peepal (*Ficus religiosa*). Those NPs’ antibacterial actions were tested on *Pseudomonas aeruginosa*. It is also obvious from the aforementioned study that raising the level of NPs increased the level of antibacterial activity. Furthermore, the nanoparticles synthesized through green synthesis have very low chances of unwanted side effects. *Ficus religiosa*, also known as the divine tree, has the potential to prepare nanoparticles, and, due to the presence of various phytochemicals, it has other benefits too. Das et al. used *Bauhinia purpurea* extraction to make MgO NPs as an antibiotic against *Staphylococcus aureus* (*S. aureus*) [4]. The antibacterial property of MgO was investigated using a fluorescence microscope as well as a scanning electron microscope. The results showed that the MgO produced had high activity as an antioxidant and antibacterial agent against *S. aureus* with a small dose (250 µg/mL). The application using the free radical 2,2-Diphenyl-1-picrylhydrazyl (DPPH), which would be extensively used during assessment of the prospects of substances to function as FRS, is a quick, easy, and affordable approach for determining antioxidant properties. Every evaluation of antioxidants as well as phytochemical capabilities of plant extraction should include an anti-free-radical activity analysis using DPPH [15,16,17]. When contrasted with ascorbic acid, the antioxidant properties of 71.72 ± 0.56% *M. oleifera* leaf aqueous extraction show excellent DPPH inhibition of 64.60 ± 0.69% RSA (radical scavenging activity). Amrulloh et al. investigated the bioactivity of MgO NPs made from aqueous *M. oleifera* bark extract [12,18]. Applying SEM and TEM pictures as well as PSA data, the average size of the particles that produce MgO NPs was 60–100 nm. The obtained MgO NPs have strong antibacterial as well as antioxidant properties against *E. faecalis*, *S. aureus*, *E. coli*, and *S. dysenteriae* bacteria. Throughout this research, we focused in this research on the classic approach using DPPH radical scavenging activities in determining *F. religiosa* antioxidant properties with green mediated MgO NPs.

## 2. Materials and Method

### 2.1. Materials

A-grade material was used, such as concentrated magnesium sulfate heptahydrate. 1,1-diphenyl-2-picryl hydrazyl (DPPH), fresh leaf extraction of *Ficus religiosa*, distilled water (DL), methanol, acetone, phosphotungstomolybdic, ferric chloride (FeCl3), ferrocyanide, gallic acid solution, etc.

### 2.2. Methods

#### 2.2.1. Preparation of Plant Extract

**Figure 1:**
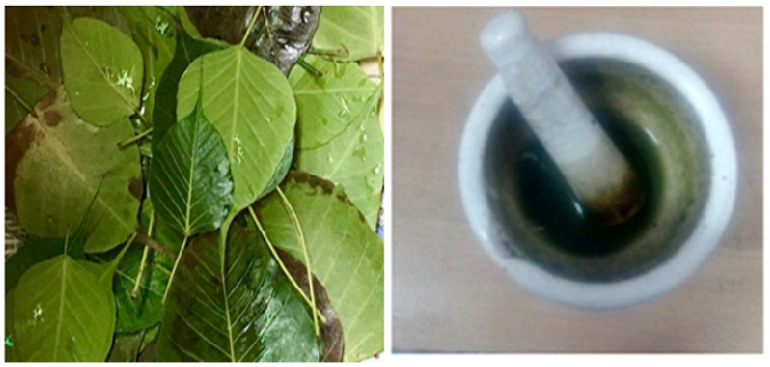
Leaves of *F. religiosa* and its Crude plant extract.

The samples were first visually examined for any kind of infection, spores, damage, discoloration, and distortion. Undamaged samples of leaf were thoroughly washed with tap water, then rinsed using DL, and then the leaves were left to dry in the light at room temperature (37 °C) The midribs of leaves were removed. Leaves and fruit were properly crushed using a mortar and pestle (Figure 1). All four samples (collected from different trees of *Ficus religiosa*) were mixed with DL, with a specimen-to-DL ratio of 1:5. In various test tubes, various samples were strained using Whatman No. 1 filter paper. To improve the quality of the filtration, certain samples were centrifuged. Some extracts were centrifuged to obtain a better-quality filtrate. Some parts of the extract were stored for further studies in an Eppendorf tube at 4 °C and the remaining extract was further diluted, filtered and stored for phytochemical analysis.

#### 2.2.2. Preparation of Green Nano-MgO Using Ficus Religiosa

Take a 15 gm leaf and crush it properly. Then add 30 mL of DL and put it into the glass beaker. Incubate the mixture for 15–20 min at 60 °C then cool it properly and filter the extract in the flask. Drop all the filtered samples into 50 mL of 0.1 M concentrate magnesium sulfate heptahydrate while stirring continuously. After incubating the specimen for 12 h, centrifuge it for 10 min at 7000 rpm. After that, discard the supernatant and wash the pellet with water.

#### 2.2.3. DPPH RSA Was Used to Test the Antioxidant Property of *F. religiosa* Dilute Leaf Extracts

The free RSA of the dilute leaf extract of *F. religiosa* was tested using a 1,1-diphenyl-2-picryl hydrazyl (DPPH) technique. A total of 24 milligrams of DPPH were dissolved in 100 mL of methanol for making the stock solution. Filtration of DPPH stock solution using methanol yielded a usable mixture with an absorbance of around 0.973 at 517 nm. In a test tube, 3 mL DPPH workable solutions were combined with 100 µL of leaf extract. Three milliliters of solution containing DPPH in 100 µL of methanol is often given as a standard. After that, the tubes were kept in complete darkness for 30 min. The absorbance was therefore determined at 517 nm. The following formula was used to compute the percentage of antioxidants or RSA [19]:

% of antioxidant activity= [(Ac−As) ÷Ac] × 100

where: Ac—Control reaction absorbance; As—Testing specimen absorbance.

#### 2.2.4. Tannin Estimation Using the Van Burden and Robinson Methods

Tannin-like substances in a strong mixture of alkaline convert phosphotungstomolybdic acid generate a bright blue solution whose intensity is related to the volume of tannins.

Use 1.6221 g of (0.1 M) ferric chloride (FeCl_3_) with 100 mL DL as a solution. Place 0.338 g (0.008 M) potassium ferrocyanide (K_4_Fe(CN)_6_) in 100 mL of DL. 0.1 N HCl: 859 µL HCl. Standard Solution of Tannic Acid: 0.05 g of tannic acid (1 mg/mL) dissolved with 50 mL of DL.

In the process, we put 20, 40, 60, and 80 g/mL of workable solutions of tannic acid from the standard inside four test tubes and then filled them with DL to make up the quantity of 5 mL. The empty test tube contains 5 mL of DL. In every test tube, make an addition of 2 mL of solution 1 and measure absorbance after 10 min. Determine the absorbance of each specimen at 605 nm using a spectrophotometer. With an empty solution, make the absorbance 0. Make a standardized graph of calibration by plotting a graph with dosage on the *x*-axis and absorbance on the *y*-axis. The slope value was applied to the graph, and then the quantity of tannic acid was calculated from an unknown specimen.

#### 2.2.5. Folin–Ciocalteu Technique for Calculating Total Phenolic Concentration

Phenolics have a broad range of biochemical properties, including being antioxidants, anticarcinogenic, antimutagenic, and having the potential to change expression of genes. Phytochemicals, the most important class of phenolics, are required for most antioxidant actions in plants and plant derivatives. The FCR (Folin–Ciocalteu reagent), commonly known as GAE (gallic acid equivalence) technique, is a colorimetric in vitro test of phenolic as well as polyphenolic antioxidants composed of phosphotungstate and phosphomolybdate.

Solution of gallic acid (0.1 mg/mL): in a glass beaker, 0.1 milligram of gallic acid was prepared by dissolving 1 mL of DL. FC solution: 0.2 N (1 mL of FC solution + 9 mL of DL). A total of 7.5% of Na_2_CO_3_ (Sodium carbonate): 15 g of Na_2_CO_3_ in 200 mL of DL.

Standardized gallic acid (0, 0.2, 2, 4, 10 g/mL) was placed into sterile test tubes as part of the protocol. To obtain the amount to 500 µL, DL was added. For every test tube, apply 2.5 mL of Folin–Ciocalteu solution, followed by incubation for 5 min at normal temperature after thoroughly mixing the solutions. Within every test tube, apply 2 mL of 7.5% Na_2_CO_3_ and incubate for 1 h at room temperature. A spectrophotometer was used to determine the absorbance of every specimen at 765 nm. With an empty solution, adjust absorbance to 0. Establish the standard calibration graph by plotting a graph using protein content on the *x*-axis and absorbance on the *y*-axis. Applying the value of slope of the graph, determine the quantity of phenols within undetermined specimens.

#### 2.2.6. Dinitrosalicylic Acid (DNS) Technique for Carbohydrate Analysis or Sugar Reduction

DNSA is lowered to 3-Amino, 5-Nitrosalicylic acid using the solution of alkaline that absorbs sunlight effectively at 540 nm and alters color from yellow to orange and reddish brown depending on the quantity of reducing sugar available in the specimen (Miller, 1959). The level was estimated using the absorbance detected in a spectrophotometer. A solution of 60% Sodium Potassium is required for the solution of tartrate: Dilute 75 mL of DL + 45 g of sodium potassium tartrate. 2 M NaOH: Dilute 1.5 g of sodium hydroxide in 50 mL of DL 3. 5-DNS solution (5%): 1.5 g DNS solution, dissolved in 30 mL of 2 M NaOH. Solution of DNS: Create a new solution combining solutions of both (a) and (b) and using DL to bring the total volume to 150 mL. Dextrose or standard solution: Dissolve 0.05 g of dextrose with 50 mL of DL to obtain a 1 mg/mL solution of dextrose.

In this procedure, we have taken clean and dry test tubes. Standard solutions in the range of 0.1 to 2 mL (as shown in the table) in five different test tubes were pipetted out and the volume of each test tube was made up to 3 mL by adding DL. Test tubes were labeled with T1, T2, T3, T4 and T5 appropriately. In another test tube, labeled as empty, add 3 mL of DL. In the leftover test tubes, 100 μL of specimen extraction as well as 2.9 mL of DL was applied to each and labeled correspondingly. Then, 2 mL of DNS solution was applied to every test tube and then they were heated in boiling hot water in a steam bath for around 5–10 min and afterwards allowed to cool. Absorbance (OD) measurements of every test tube were collected at 540 nm using a spectrophotometer (Shimandzu, Tokyo, Japan). The outcome of multiple standard solutions thus generated has been used to draw calibration curves containing carbohydrates, and the content of carbohydrates in undetermined specimen extraction) was evaluated.

#### 2.2.7. Folin and Lowry Methods for Total Protein Content Estimation

According to this approach, protein content may be determined using alkaline reactions between copper ions as well as the peptide nitrogen, which then reduces Folin–Ciocalteu Phosphomolybdic Phosphotungstate acid into heteropolymolybednum blue through copper-catalyzed oxidation of aromatic acid. Because of the method’s sensitivity to pH, it must be kept between 10 and 10.5 at all times. A total of 2% Na_2_CO_3_ solution is needed as a solution of 00 mL of DL, 2 g Na_2_CO_3_, and 0.4 g NaOH (0.1 N NaOH) dissolved. Sodium potassium at a concentration of 1% solution of tartrate: Use 2 mL of DL to dilute 0.02 g sodium potassium tartrate. Solution of CuSO_4_ at a concentration of 0.5%: Use 2 mL of DL to dilute 0.01 g of copper sulfate. Solution 1: Mix a solution of 96 mL (a), 2 mL solution (b) and 2 mL solution (c). Solution 2: Dissolve 2.5 mL 2N Folin–Phenol in 2.5 mL of DL. Standard solution, i.e., bovine serum albumin (BSA): 2.5 mL 2N Folin–Phenol dissolved in 2.5 mL of DL (1 mg/mL).

Standard BSA (0, 30, 60, 120, 240, and 480 μL) was placed in 5 thoroughly clean and dry test tubes and were labeled blank, T1, T2, T3, T4, and T5. To continue making the 1000 μL, DL was added. In another test tube, 25l μL of extract solution and 975 μL of DL were mixed and labeled. Every test tube contained 4.5 mL of solution 1 and was kept for around 10 min at room temperature. Every specimen solution was therefore filled with 500 μL of solution 2 and left to sit at room temperature for 30 min. Every test tube absorbance was measured using a spectrophotometer at 660 nm. A standard curve for proteins was plotted using the results of several standard solutions, and the protein content in unknown factors (extracted from samples) was approximated.

All plots can be calculated as:

y = mx + c or x = (y − c)/m

## 3. Results

Magnesium oxide nanoparticles have a large band gap, good physicochemical qualities, and a lot of surface area. They are said to have high antibacterial capabilities as a result of these features. Furthermore, when MgO nanoparticles come into contact with sufficient oxygen in the bacterial cell wall, they tend to generate super oxides. The super oxides formed are highly reactive, destroying the cell wall of bacteria and phospholipids within it extremely quickly. The discovery of antioxidant properties in a dilute extract of leaves of *F. religiosa* using DPPH RSA are addressed in this part. The Van Burden and Robinson method for tannin determination, the Folin–Ciocalteu system for estimation of overall phenolic level, the Dinitrosalicylic Acid (DNS) technique for estimating carbohydrate or reducing sugar analysis, and the Folin and Lowry technique for total protein content estimation are as follows:

When free-radical DPPH interacts with an odd electron, the greatest absorption takes place at 517 nm (purple color). A free-radical scavenger antioxidant reacts to DPPH to form DPPHH, which has a lower absorbance than DPPH because of the lower amount of hydrogen. In comparison to the DPPH-H state, this radical version causes decolorization (a yellow hue) as the number of electrons collected increases.

**Figure 2:**
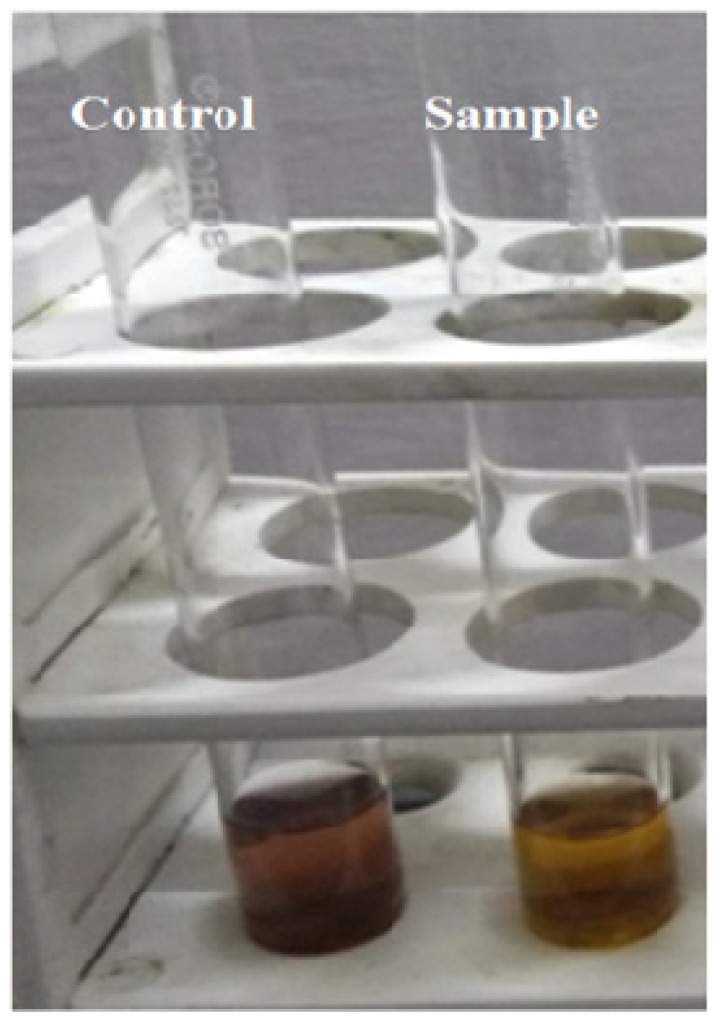
Antioxidant activity of *F. religiosa* with DPPH.

It can be observed from Table 1 and Table 2 that the antioxidant activity of *F. religiosa* showed 0.550 absorbance at 517 nm with the percentage of antioxidant as 43.415% and MgO nanoparticles showing absorbance of 0.461 at 517 nm with the percentage of antioxidant as 57.783% with DPPH. After nano formation, antioxidant activity increases (Figure 2).

### 3.1. Tannin Estimation

**Figure 3:**
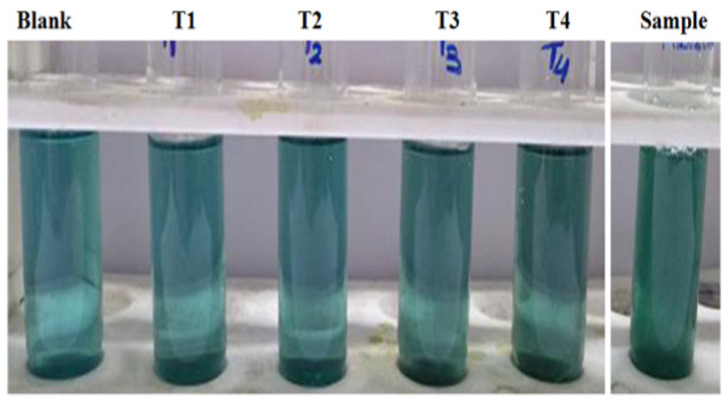
Tannin estimation by tannic acid standard.

**Figure 4:**
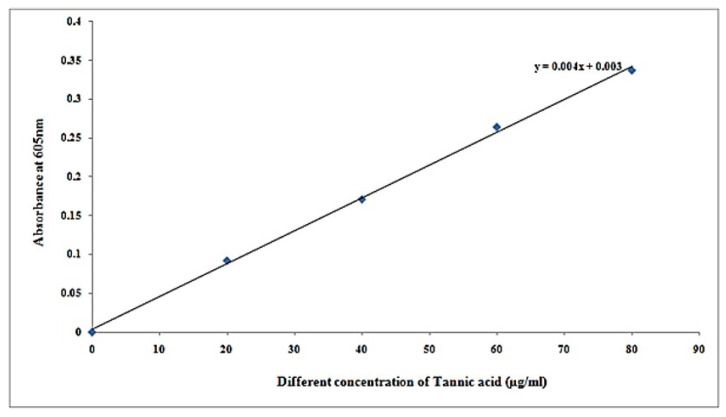
Standard curve of tannic acid.

It was found that tannin estimation from different samples, as shown in Table 3 and Table 4 with Figure 3, showing coloration absorbance effect was found to increase with increasing absorbance plot, as shown in Figure 4.

It can be seen from Table 5 that tannin concentration in samples at concentration of µg/500 µL was 74.75, and concentration of µg/mL is 149.5 at 0.302 absorbance at 605 nm.

### 3.2. Total Phenolic Content Estimation

**Figure 5:**
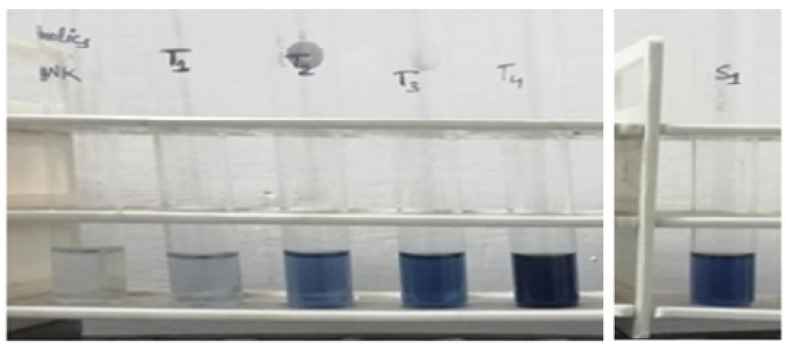
Specimen resemblance of total phenolic concentration.

**Figure 6:**
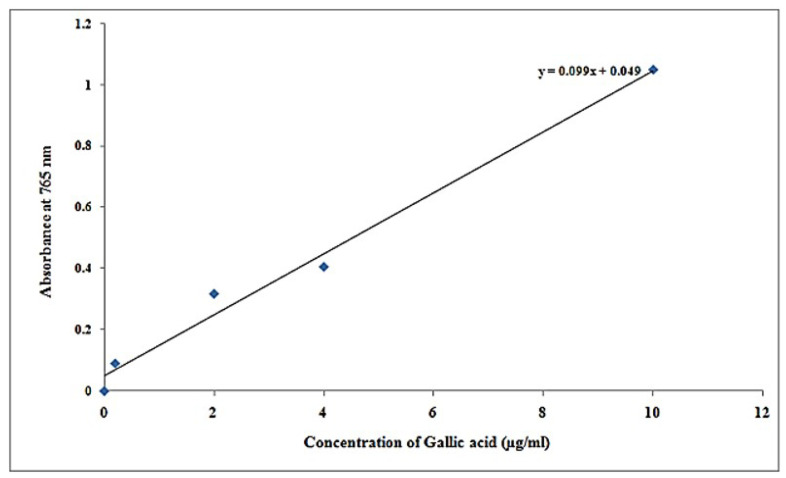
Graph plot of gallic acid results.

It was observed that total phenolic content estimation in different concentrations of specimen by FC method in Table 6 and its specimen resemblance of total phenolic content in Figure 5 shows darkened coloration due to the increased concentration standard (gallic acid). Table 7 displays the results, and Figure 5 depicts the plot. *x* = concentration, *y* = absorbance, c = intercept and m = slope, respectively (Figure 6).

It was found that phenolics concentration in samples at concentration of µg/100 µL was 5.11, and at concentration of µg/mL is 51.11 at 0.555 absorbance at 765 nm (Table 8).

### 3.3. Estimation of Carbohydrates or Reducing Sugar

**Figure 7:**
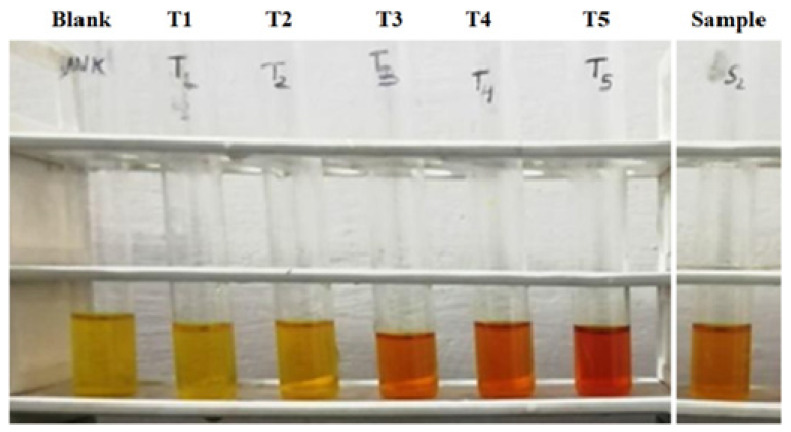
Reducing sugar estimation by DNS method.

Table 9 shows the estimation of carbohydrates or reducing sugar with different specimen formulations by the DNS method and the color resemblance of reducing sugar estimation, showing darkening with increasing concentration of sugar concentration and absorbance of blank and standard solution dextrose at concentration of µg/mL) with a range of 0–499.95 in Table 10, and its respective plot shown in Figure 7 respectively. It is shown that concentration increases with respect to absorbance.

It was discovered that after lowering the sugar concentration in various samples at 0.292 absorption at 540 nm, the concentration of µg/100 l is 269 and the concentration of µg/mL is 2690 (Table 11).

### 3.4. Estimation of Protein Concentration

**Figure 8:**
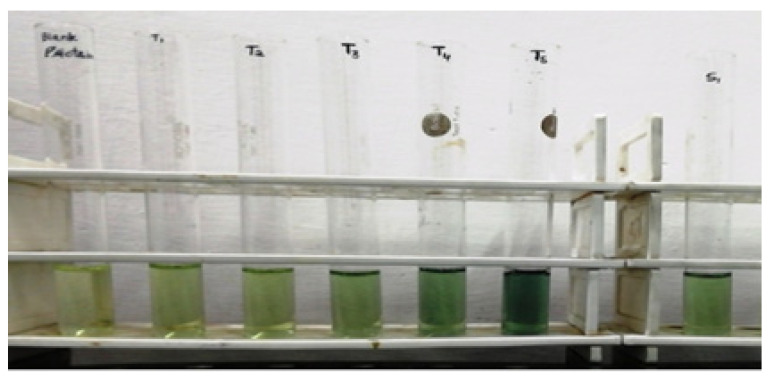
Specimen resemblance of protein concentration.

**Figure 9:**
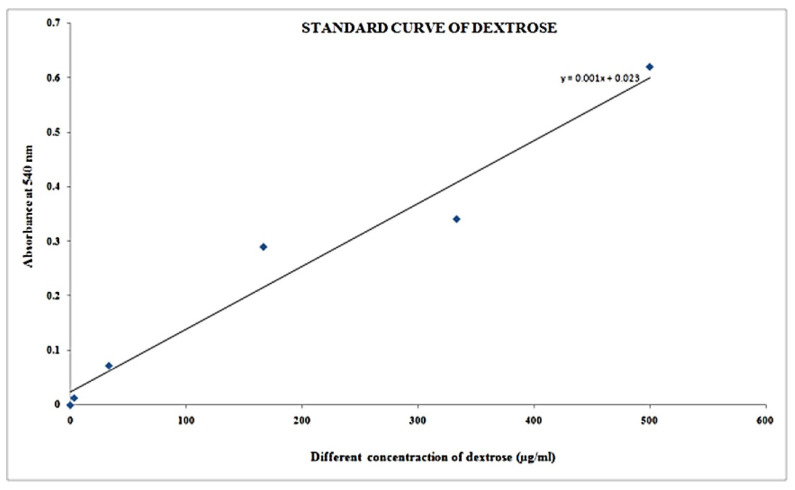
Plot Concentration vs. absorbance.

**Figure 10:**
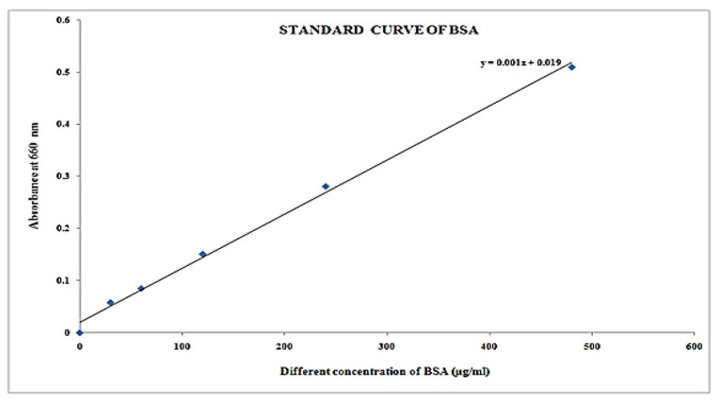
Graph of concentration versus absorbance of blank and standard solution.

It was found that protein at different concentrations of specimen by the Lowry method presented in Table 12 and specimen resemblance of protein concentration presented in Figure 8 was green, and darkening with increasing concentration of protein. Its absorbance of blank and standard solutions is given in Table 13 and its respective plot of concentration versus absorbance is presented in Figure 9. The same trends are observed here, with protein concentration increasing in relation to absorbance (Figure 10).

It was found that protein concentration in different samples from Table 14 at concentration of µg/25 µL is 263, and concentration of µg/mL is 10,520 at 0.282 absorption at 660 nm, respectively.

## 4. Discussion

Plants have long been known to have anticancer effects. Increased unfavorable negative impacts induced by several cancer chemotherapeutic treatments may have been the primary motivator for using alternative therapies with the aim of finding a better and safer cure for cancer [33] The majority of people throughout the world currently use herbal remedies as part of their medical system, indicating that plant-based traditional medicine will continue to play an important role in human healthcare in the coming years. Growing expenditures on prescribed drugs to preserve good health and well-being have revived interest in traditional medicines in healthcare. Medicine discovered from plants might be less expensive, have low toxicity, or even be toxic-free due to bioprospecting [34].

The free radical DPPH, which is widely used to evaluate the ability of compounds to operate as free-radical scavengers and hydrogen suppliers, is a rapid, simple, and inexpensive method for testing antioxidant capabilities. The DPPH test relies on the elimination of DPPH, a stabilized free radical. DPPH is indeed a dark-colored crystalline compound made up of free-radical particles that are stable. In particular, it is a well-known radical and a popular antioxidant test. Once, reduced and transformed into DPPH-H, the DPPH radical has a dark purple hue in solution, but when reduced as well as transformed into DPPH-H, it turns colorless or light yellow [35]. In vitro, several extractions of plants have been shown to neutralize DPPH radical scavenging activity [36,37,38,39,40]. DPPH radicals were scavenged by various extracts of *F. religiosa* in a content-dependent approach. Furthermore, DPPH free radicals have been found to be scavenged by several tea extracts consisting of a variety of polyphenols [41]. DPPH free-radical scavenger kaempferol, found in a variety of species, particularly *F. religiosa*, has a value of IC50 estimated to be around 0.004349 mg·mL^−1^ [42,43,44]. Mangiferin, as well as naringin, for example, have been shown to scavenge DPPH radicals in a content-dependent way [37,45]. The activity of scavenging of *F. religiosa* ethanol extraction indicated 80 and 140 g/mL for aqueous extraction, respectively, and doubles the dosage of ethanolic extracts (160 g/mL) to extract chloroform. Using DPPH, we discovered in this study that *F. religiosa* has antioxidant properties of 0.550 absorbency at 517 nm with the proportion of antioxidants as 43.415%, while MgO NPs had an antioxidant property of 0.461 at 517 nm with a proportion of antioxidant of 57.783%. For tannin concentrations in multiple specimens with 0.302, the level (µg/500 µL) is 74.75, and the content (µg/mL) is 149.5. For phenolics content with varied absorption at 605 nm at 0.555, the content (µg/100 µL) is 5.11, while the content (µg/mL) is 51.11 for 765 nm. Reducing the content of sugar with concentration of µg/100 µL is 269 and concentration of µg/mL is 2690 with 0.292 absorbency at 540 nm. Protein content with concentration of µg/25 µL is 263 and concentration of µg/mL is 10,520 with 0.282 absorbency at 660 nm. As a result, a significant DPPH scavenging action of *F. religiosa* could be related to the existence of flavonoids as well as other polyphenols throughout extraction, as shown in the current work.

Hydroxyl radicals are very reactive and have a limited lifespan [46]. They can cause harm to essential macromolecules such as nucleic acids and proteins. Hydroxyl radicals are formed by hydrogen peroxide throughout the context of iron ions within Haber–Weiss/Fenton process [47,48]. The extreme sensitivity of hydroxyl radicals causes significant harm to cells and their elements, as well as to organisms as a whole [49]. As a result, removing hydroxyl radicals that have negative consequences is crucial. The production of hydroxyl free radicals was reduced by various extracts of *F. religiosa* in a content-dependent approach. In a previous work, the flavonoid kaempferol found within *F. religiosa* scavenged OH radicals [42]. Numerous extractions of plants and flavonoids, such as mangiferin and naringin, have also been shown to scavenge hydroxyl free radicals in a content-dependent approach [35,36,37,40,45]. Numerous flavonoids generated as secondary metabolites from various plants have previously been demonstrated to scavenge OH radicals [50,51].

Because O_2_^•−^ is produced in the biological system during cell respiration, it is less hazardous; however, in the presence of iron, it is transformed into a strongly reactive OH radical [52]. In addition, superoxide anions formed by inadequate oxygen metabolism harm biomolecules explicitly or implicitly by producing H_2_O_2_, ^•^OH, peroxynitrite, or singlet oxygen [52,53]. As a result, superoxide radicals must be removed or neutralized in terms of protecting cells from their detrimental consequences. The production of O_2_^•−^ was suppressed by several extracts of *F. religiosa* in a content-dependent approach. In a previous study [42], kaempferol was discovered to scavenge O_2_^•−^. Naringin, mangiferin, myricetin, quercetin, and rutin, among other extractions of plants and flavonoids, have been discovered to scavenge superoxide free radicals in a content-dependent approach [35,36,37,40,45,54]. Various in vitro approaches may be used to measure the antioxidant properties (total antioxidant potential) of plants as well as plant products. There have been 2 kinds of assays that are often applied in antioxidant investigation. The DPPH test, Trolox equivalent antioxidant capacity (TEAC) test, and FRAP test are three of the first sets of experiments linked to electrons or radical scavenging. They work based on the reduction process. The thiobarbituric acid test and the β-carotene bleach experiment (Moon & Shibamoto, 2009) are two examples of lipid peroxidation studies [55]. The DPPH test is used to estimate antioxidant activity based on the process through which antioxidants limit lipid oxidation, resulting in DPPH free-radical scavenging and therefore determining free-radical scavenging potential. The approach has been extensively used since the analysis takes just a few minutes. The DPPH free radical is extremely stable, reacts with hydrogen chemicals, and has a UV–vis absorbance maxima of 515 nm. The approach relies on antioxidants scavenging DPPH, which decolorizes the solution of DPPH methanol after a reduction process. The antioxidant capacity to reduce the DPPH radical is measured in this test. Total radical scavenging capability is also measured using the TEAC test. The test depends on the discoloration of a formed ABTS (2,2′-azinobis-3-ethylbenzothiazoline-6-sulfonic acid) radical with antioxidant chemicals, which reflects the quantity of ABTS radicals scavenged in comparison to Trolox during a set timeframe (6-hydroxy-2,5,7,8-tetramethylchroman-2-carboxylic acid). By comparing the reduction throughout absorbency to Trolox absorbance at 734 nm, the total radical scavenging capability of the specimen can be estimated. The value of TEAC represents the capacity of test samples to respond with the ABTS radicals instead of inhibiting the process of oxidation, which is a shortcoming of this approach. Plant extraction antioxidant activity is measured by the capacity to convert Fe^3+^-tripyridyltriazine into Fe^2+^-tripyridyltriazine using the FRAP (ferric reducing antioxidant properties) method. The testing is performed on electron-transfer processes wherein potassium ferricyanide, a ferric salt, is applied as an oxidant. The oxidation of ferric 2,4,6-tripyridyl-s-triazine to the colorful ferrous state is the reaction mechanism. ROS generation can be increased by defects in oxidative phosphorylation, and ROS-mediated degradation to biomolecules can have real implications on electron transport system components [56,57]. The frequency of absorbance is 593 nm. The fluorescent β-phycoerythrin (B-PE) is used as an oxidizing protein substrate (probe) while AAPH (2,2′-azobis (2-amidinopropane) dihydrochloride) is used to create peroxyl radicals in the ORAC (oxygen radical absorbance capacity) experiment. Furthermore, under fluorescent plate-reader settings, B-PE is photobleached and interacts with phenolic chemicals because of non-specific protein binding. The addition of antioxidants to the mixture interacts with the substrates for peroxyl radicals, preventing or delaying fluorescein oxidation. In the existence of peroxyl radicals created at a regulated pace through heating breakdown of AAPH inside an air-saturated solution, the substrates (fluorescein) decompose. The fluorescence emission is recorded in a range of 525 nm for discharge and 485 nm for extinction. Fluorescein emission intensity falls as it is used in ORAC reactions. Ndhlala et al. (2010) [34] used the ORAC test to determine both hydrophilic as well as lipophilic chain-breaking antioxidant ability. Thiobarbituric acid-reactive compounds have been used to prevent lipid peroxidation in the lipid peroxidation inhibition capability test. In addition, new techniques for determining antioxidant properties are continually being developed. Ivanova, Brainina, Lozovskaya, Sharafutdinova, and Shkarina (2007) developed a potentiometry approach that correlates well with various standard antioxidant activity measurement methodologies (DPPH, TEAC) [58,59].

The specific method of free-radical scavenging using various *F. religiosa* extraction is unknown. Furthermore, phytochemical study of *F. religiosa* stem barks reported the existence of phenolic and flavonoid compounds, which rose in content as the quantity of extraction rose. As a result, the existence of multiple polyphenols as well as flavonoids within *F. religiosa* may be responsible for free-radical scavenging as well as antioxidant properties. Free-radical scavenging as well as antioxidant properties of *F. religiosa* as well as its produced nano-MgO may be due to the presence of certain key components such as carbohydrates, tannin, phenolics, reducing sugar, protein substances, and flavonoids.

## 5. Conclusions

We demonstrated in this study that a free radical is applied in the 2,2-Diphenyl-1-picrylhydrazyl (DPPH) technique for measuring the capacity of substances to serve as scavenging free radicals or hydrogen suppliers, which can be a quick, easy, and affordable approach for determining antioxidant properties. The decrease of DPPH, a stable free radical, is used in the DPPH analysis technique. The DPPH free radical interacts with an odd electron to produce an absorption maximum wavelength of 517 nm (purple color). Antioxidants combine with DPPH and combine the existence of a hydrogen source (for example, a free-radical scavenging antioxidant), resulting in the reduction of DPPH to DPPHH and a reduction in DPPH absorbency. In contrast to the DPPH-H form, decolorization (i.e., a yellow hue) occurs as the number of electrons collected increases. The ability of decolorization is related to decreasing ability. Whenever a DPPH solution is combined with a hydrogen atom giving material, the reduced state, diphenylpicrylhydrazine (non-radical), is formed, and the violet color is lost (despite the fact that a residual light yellow color from the picryl group should still be apparent).

Our study reveals that all *F. religiosa* extracts inhibited free radicals and boosted decreasing antioxidant capacity in a content-dependent approach. The inclusion of different tannins, phenolics, carbohydrates or reducing sugar, components of protein, and flavonoids, among other things, may be responsible for *F. religiosa* activity. The antioxidant properties of the ethanolic extracts were highest, then the aqueous leaf extract, and the chloroform extract was lowest. Our research found that *F. religiosa* has antioxidant properties and might be effective in the treatment of free-radical-induced diseases.

Antioxidants aid in the neutralization of free radicals, the primary cause of inflammatory conditions, and thus may try to protect from diseases caused by free radicals. The use of *F. religiosa* might be beneficial in inflammatory illnesses and can be used for a variety of health conditions. Furthermore, further research on *F. religiosa* will be needed to identify its active ingredients for medical application. The goal of activity-guided extraction of various phytochemicals is to determine their antioxidant capacity as well as other disease-curing abilities using various preclinical studies. Our study contributes to providing information through this investigation on *F. religiosa*, where all antioxidant properties found are extremely effective in treating free-radical-induced diseases such as cardiovascular and inflammatory diseases, cancer etc.

## Figures and Tables

**Table 1:** Antioxidant activity of *F. religiosa* with DPPH.

S. No.	Absorbance at 517 nm	% of Antioxidant
Control	0.972	-
Sample	0.550	43.415

Calculation of Antioxidant activity of *F. religiosa* with DPPH; % of antioxidant activity = [(Ac − As) ÷ Ac] × 100; % of antioxidant activity = [(0.972 − 0.550) ÷ 0.972] × 100; % of antioxidant activity = 43.415.

**Table 2:** Antioxidant activity of green synthesis of MgO nanoparticles with DPPH.

S. No.	Absorbance at 517 nm	% of Antioxidant
Control	1.092	-
MgO nanoparticles	0.461	57.783

Calculation of Antioxidant activity of green synthesis of MgO NPs with DPPH; % of antioxidant activity = [(Ac − As) ÷Ac] × 100; % of antioxidant activity = [(1.092 − 0.461) ÷ 1.092 × 100; **%** of antioxidant activity = 57.783.

**Table 3:** Estimation of tannin from a specimen.

**Tube**	**d H_2_O (mL)**	**Standard/Specimen (mL)**	**Reagent 1 (mL)**	Incubate at RT for 10 min	Take the O.D after 10 min at 605 nm
Blank	5	0	2		
T1	4.9	0.1	2		
T2	4.8	0.2	2		
T3	4.7	0.3	2		
T4	4.6	0.4	2		
Sample	4.5	0.5	2		

Sample = *Ficus religiosa*.

**Table 4:** Tannic acid content and absorbance.

Tube	Content (µg/mL)	Absorbance at 605 nm
Empty	0	0.000
T1	20	0.092
T2	40	0.171
T3	60	0.264
T4	80	0.337
Specimen (*Ficus religiosa)*	-	0.302

**Table 5:** Tannin concentration in samples.

Test Tube	Absorbance at 605 nm	Concentration (µg/500 µL)	Concentration (µg/mL)
Specimen	0.302	74.75	149.5

(Specimen = *Ficus religiosa*).

**Table 6:** Determination of total phenolic concentration in a specimen using the FC technique.

**Specimen**	**d H_2_O** **(µL)**	**GA/Specimen** ** (µL)**	**FC Reagent** **(mL)**	Incubate at room temp. for 5 min.	**7.5 % Na_2_CO_3_** **(mL)**	Incubate at room temp for 1 h	Absorbance at 765 nm
Blank	500	0	2.5		2		
T-1	499	1	2.5		2		
T-2	490	10	2.5		2		
T-3	480	20	2.5		2		
T-4	450	50	2.5		2		
T-5	400	100	2.5		2		
Sample 1	400	100	2.5		2		

**Table 7:** FC standard result (gallic acid).

Specimen	Concentration of Gallic Acid (µg/mL)	Absorbance at 765 nm
Blank	0	0.000
T-1	0.2	0.090
T-2	2.0	0.318
T-3	4.0	0.406
T-4	10.0	1.050
Specimen	-	0.555

**Table 8:** Phenolics concentration in samples.

Test Tube	Absorbance at 765 nm	Concentration (µg/100 µL)	Concentration (µg/mL)
Sample	0.555	5.11	51.11

(Specimen = *Ficus religiosa*).

**Table 9:** Estimation of reducing sugars (by DNS method).

**Test Tube**	**DL** **(mL)**	**Standard/Sample** **(mL)**	**DNS Reagent** **(mL)**	Incubate in boiling water bath for 5–10 min and allow to cool	Take O.D. at 540 nm
Blank	3	0	2		
T_1_	2.9	0.1	2		
T_2_	2.5	0.5	2		
T_3_	2	1	2		
T_4_	1.5	1.5	2		
T_5_	1	2	2		
Specimen 1	2.9	0.1	2		

**Table 10:** Empty and standard solution concentrations.

Test Tube	Absorbance at 540 nm	Dextrose Concentration (µg/mL)
Blank	0.000	0.000
T1	0.013	3.33
T2	0.072	33.33
T3	0.290	166.65
T4	0.341	333.33
T5	0.620	499.95
Specimen (*Ficus religiosa)*	0.292	

**Table 11:** Reducing sugar concentration in different samples.

Test Tube	Absorbance at 540 nm	Concentration (µg/100 µL)	Concentration (µg/mL)
Sample	0.292	269	2690

**Table 12:** Quantification of protein by Lowry method.

**Test Tubes**	**BSA/Specimen** **(µL)**	**DL** **(µL)**	**Reagent 1** **(mL)**	Incubation at room temperature for 10 min	**Reagent 2** **(µL)**	Incubation in dark for 30 min	Take OD 660 nm
Blank	0	1000	4.5		500		
T1	30	970	4.5		500		
T2	60	940	4.5		500		
T3	120	880	4.5		500		
T4	240	760	4.5		500		
T5	480	520	4.5		500		
Specimen	25	975	4.5		500		

**Table 13:** Empty and standard solution contents and absorbance.

Test Tube	BSA Content (µg/mL)	Absorbance at 660 nm
Blank	0.000	0.000
T1	30	0.058
T2	60	0.085
T3	120	0.181
T4	240	0.281
T5	480	0.510
Specimen	-	0.282

**Table 14:** Protein concentration in different samples.

Test Tube	Absorbance at 660 nm	Concentration (µg/25 µL)	Concentration (µg/mL)
Specimen	0.282	263	10,520

## Data Availability

Not applicable.

## Sample Availability

Samples of the compounds are available from the authors.
